# Biomimetic core–shell nanofibrous scaffolds with an organic montmorillonite interlayer for tissue engineering

**DOI:** 10.3389/fbioe.2026.1836027

**Published:** 2026-05-14

**Authors:** Xiangrui Mao, Ruobing Cheng, Jian Zhang, Yuxiang Yao, Nan Jiang, Jinbiao Zhang, Tanlong Liu, Zhirang Hu, Yutong Liu, Zhiyong Yan, Chengwei Yang, Anlin Yin, Kuihua Zhang

**Affiliations:** 1 College of Materials and Textile Engineering, Jiaxing University, Jiaxing, Zhejiang, China; 2 Analytical and Testing Center, Jiaxing University, Jiaxing, Zhejiang, China; 3 College of Materials Science and Engineering, Hunan University of Technology, Zhuzhou, China; 4 Department of Orthopaedics, The 940th Hospital of Joint Logistics Support Force of PLA, Lanzhou, Gansu, China

**Keywords:** antibacterial, core-shell nanofiber with OMMT interlayer, degradation, silk fibroin, tissue engineering

## Abstract

Electrospun polymer nanofibrous scaffolds have gained considerable interest in biomedical applications due to their ability to mimic the architecture and biological functions of the extracellular matrix (ECM). However, precise control over scaffold degradation is essential for ensuring predictable material behavior and supporting their intended function in tissue engineering. In this study, we develop a series of core–shell nanofibrous scaffolds composed of glycidyl methacrylate-grafted silk fibroin and poly(L-lactide-co-caprolactone) (SFMA/PLCL), incorporating an interlayer of organically modified montmorillonite (OMMT). The introduction of OMMT markedly enhances scaffold hydrophilicity, mechanical robustness, and dimensional stability, while enabling finely tunable degradation kinetics. Increasing OMMT content shifts the degradation mode from surface erosion to bulk degradation by facilitating water penetration and disrupting polymer interactions through exfoliated OMMT nanosheets and the release of quaternary ammonium ions. Moreover, the scaffolds exhibit potent antibacterial activity (>99% inhibition against Staphylococcus aureus), strong anti-biofilm performance (>75% suppression), and long-lasting antibacterial effects, all while maintaining excellent cytocompatibility. These results demonstrate that core–shell nanofibrous scaffolds with an OMMT interlayer represent a promising multifunctional platform for tissue engineering applications.

## 1 Introduction

Electrospun polymer nanofibers have been extensively employed in biomedical applications, such as tissue engineering scaffolds, wound dressings, biosensors, and drug delivery carriers, owing to their remarkable capacity to biomimic the structural and functional characteristics of the extracellular matrix (ECM), coupled with their inherent advantages of high specific surface area and interconnected porous architecture (Abboud et al., 2025; Jin et al., 2025; Yuan et al., 2025). However, a major challenge in the practical application of nanofibrous scaffolds is achieving precise control over their degradation kinetics and biofunctional performance. Scaffold degradation is triggered immediately upon implantation, with byproducts influencing cell function and immune activity. Ideally, degradation kinetics should align with tissue regeneration to support scaffold replacement by native tissue (Cui et al., 2008; Laycock et al., 2017; Tesfamariam, 2016). Degradation behavior is also central to drug and growth factor delivery, where it governs the release profile alongside diffusion gradients. By modulating degradation mechanisms, it is possible to tailor release rates and mimic physiological expression patterns, improving therapeutic outcomes (Arango et al., 2025; Sun et al., 2021; Jiao et al., 2025).

Montmorillonite (MMT), a layered silicate mineral, has attracted growing interest in biomaterials due to its high surface area, cation exchange capacity, antibacterial property and biocompatibility (Ngo et al., 2024; Yang et al., 2025). When organically modified (OMMT), it shows improved compatibility with polymers. The incorporation of MMT or OMMT generally enhances the dimensional stability, mechanical strength, thermal stability, barrier properties of polymers owing to its unique layered architecture (Luo et al., 2025; Wang et al., 2025; Zhang et al., 2025). In recent years, polymer/MMT or OMMT nanocomposite nanofibers have been extensively explored as biomaterials (Kausar et al., 2023; Sayyar et al., 2024; Karimi‐Soflou et al., 2021). Sayyar et al. (2024) reported that MMT can electrostatically interact with curcumin under various pH conditions in curcumin-loaded magnetic MMT/polyvinyl alcohol/hyaluronic acid/chitosan nanofiber mats, thereby regulating its sustained release performance and imparting pH-dependent behavior. Hamidabadi et al. (Karimi‐Soflou et al., 2021) showed that the OMMT/PVA nanofibrous artificial nerve graft promotes neural cell proliferation, migration, and differentiation more effectively than PVA nanofibers. The incorporation of OMMT not only enhanced the mechanical properties of the PLLA nanofiber scaffold but also promoted osteogenesis, as the mineral composition of MMT is similar to that of bone minerals, creating an ideal microenvironment for bone regeneration (Ghasemi Hamidabadi et al., 2017). Our previous work demonstrated that a novel silk fibroin/poly(L-lactic acid) (SF/PLLA) core-shell nanocomposite nanofiber with an OMMT interlayer exhibited superior hydrophilicity, thermal and mechanical stability, and long-lasting antibacterial performance without cytotoxicity (Gu et al., 2024). We also found that polymers or their blends soluble in HFIP, such as PCL, PLLA, Polyamide (PA), Polysulfone (PSU) Polyurethane (PU), Collagen, and so on, can be co-blended with OMMT to prepare uniform spinning solution. The core-shell structured nanofibers with OMMT interlayer can be successfully fabricated via conventional electrospinning techniques. Furthermore, the OMMT content within the nanofibers can be tuned within a certain (Zhou et al., 2025). However, their degradation behavior, especially in core-shell architectures with OMMT interlayer, remains insufficiently understood.

**SCHEME 1:**
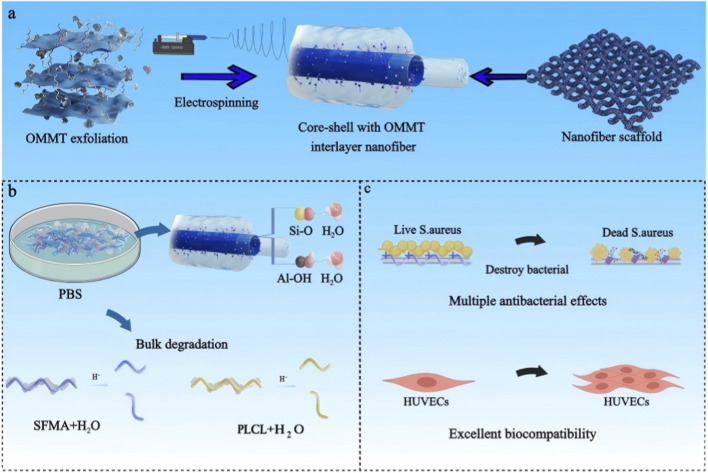
Illustration of **(a)** the preparation of core-shell with OMMT interlayer nanofibrous scaffold; **(b)** bulk degradation; **(c)** antibacterial property and biocompatibility.

Silk fibroin (SF), a natural protein from silkworm cocoons, is extensively used in biomedical materials due to its biocompatibility, biodegradability, and gas/moisture permeability (Fan et al., 2025). Blending SF with synthetic polymers like poly(L-lactide-co-caprolactone) (PLCL) improves mechanical strength and enables better-tuned degradation for regenerative applications (Zhang et al., 2010; Zhang et al., 2011). To further enhance performance, SF can be grafted with glycidyl methacrylate (GMA) to form SFMA, enabling photo-crosslinking and improved structural integrity in nanofibrous scaffolds (Fan et al., 2023). In this study, a core-shell structured SFMA/PLCL nanofibrous scaffold with a tetradecyl dimethyl benzyl ammonium-modified MMT (OMMT) interlayer was prepared using conventional electrospinning techniques (Scheme 1). The OMMT interlayer is of critical importance, as it significantly enhances the nanofibrous scaffold’s mechanical properties, hydrophilicity, mechanical strength, and dimensional stability. Additionally, the OMMT interlayer confers potent antibacterial activity (over 99% inhibition against *Staphylococcus aureus*) and robust anti-biofilm effects (over 75% suppression), while maintaining excellent cytocompatibility. Notably, the OMMT content can be adjusted to modulate the degradation rate of the nanofibrous scaffold, facilitating a shift in the degradation mechanism from surface erosion to bulk degradation. The OMMT interlayer plays a key role in controlling how the nanofibrous scaffold degrades and fights bacteria. This provides useful insights for using it in tissue regeneration, drug delivery, and other biomedical applications.

## 2 Materials and methods

### 2.1 Materials

Silkworm cocoons were provided by Jiaxing Silk Company (China). Poly(L-lactide-co-caprolactone) (PLCL) (50:50), which has a composition of 50 mol% L-lactide, was purchased from Daigao Co. Ltd. (Shandong, China). Sodium montmorillonite (Na-MMT, cation exchange capacity of 100 mmol/100 g) was supplied by Zhejiang Yuhong Clay Co., Ltd. (Zhejiang, China). Tetradecyl dimethyl benzyl ammonium chloride (TDMBACl), hexafluoroisopropanol (HFIP), and glycidyl methacrylate (GMA) were obtained from Aladdin Reagent Co., Ltd. (Shanghai, China).

### 2.2 Fabrication of nanofibrous scaffolds

The fabrication procedures for OMMT and SFMA are detailed in the Supplementary Material. SFMA and PLCL were dissolved in HFIP at a mass ratio of 8:2 to obtain an 8 wt% SFMA/PLCL blend solution. OMMT was then incorporated into the blend at concentrations of 1 wt%, 3 wt%, and 5 wt% (w/w, relative to the total mass of SFMA and PLCL), followed by thorough dissolution in HFIP to form homogeneous 8 wt% SFMA/PLCL/OMMT solutions. Electrospinning was performed under optimized conditions: a flow rate of 1.2 mL/h, an applied voltage of 15 kV, and a collector distance of 15–20 cm. Four types of nanofibrous scaffolds were fabricated and designated as SFMA/PLCL, SFMA/PLCL/OMMT-1, SFMA/PLCL/OMMT-3, and SFMA/PLCL/OMMT-5, respectively. To stabilize the nanofibrous structure, the scaffolds were exposed to 75 vol% ethanol vapor for 12 h, inducing a conformational transition of SFMA into an insoluble β-sheet configuration (Gu et al., 2024).

### 2.3 Characterization

The surface morphology of the nanofibrous scaffolds was examined using scanning electron microscope (SEM, S-4800, Japan), and their internal microstructure was analyzed via transmission electron microscope (TEM, Talos F200X, United States). High-angle annular dark-field (HAADF) images were acquired in scanning transmission electron microscopy (STEM) mode to characterize the fine structure of the nanofibers. A Super X-EDS system (including four windowless silicon drift detector (SDD) detectors) was used to perform energy-dispersive X-ray spectroscopy (EDS) mapping of the target area (pixel dwell time, 10 μs; acquisition. Fourier transform infrared spectroscopy with attenuated total reflectance (FTIR-ATR), X-ray diffraction (XRD), thermogravimetric analysis (TGA), and derivative thermogravimetric analysis (DTG) were employed to analyze the chemical structure and thermal stability of the nanofibrous scaffolds. These measurements were carried out using an FTIR spectrometer with attenuated total reflectance (Vertex 70, Germany), an X-ray diffractometer (D8-A25, Germany), and a thermogravimetric analyzer (Q50, United States), respectively. Surface wettability was assessed by static water contact angle measurements using a contact angle analyzer (DSA 100, Krüss, Germany). Wettability dynamics were evaluated by extracting still frames at defined time intervals from real-time video recordings.

### 2.4 Evaluation of nanofibrous scaffolds properties

The mechanical properties of the nanofibrous scaffolds under hydrated conditions, along with their swelling behavior, were evaluated following established protocols reported in the literature (Gu et al., 2024). The water vapor transmission rate (WVTR) was measured using the standard evaporation method outlined in ASTM E96. Detailed testing procedures are provided in the Supplementary Material.

### 2.5 *In vitro* antimicrobial and anti-biofilm assays

The Gram-positive bacterium *S. aureus (S. aureus)* was employed to evaluate the antibacterial and anti-biofilm properties of the nanofibrous scaffolds, as well as the antibacterial activity of the slow-release solution derived from SFMA/PLCL/OMMT nanofibrous scaffolds. Antibacterial performance was assessed using the shaking method, as outlined in GB/T 20,944.3–2008 and based on previous literature (Zhou et al., 2021; Miranda-Calderon et al., 2023). Detailed experimental methods and procedures are provided in the Supplementary Material.

### 2.6 Cytocompatibility evaluation

The cytocompatibility of the nanofibrous scaffolds was assessed by evaluating the proliferation of human umbilical vein endothelial cells (HUVECs; Cell Bank of the Chinese Academy of Sciences, Shanghai, China). The assays were conducted in accordance with ISO 10993–5 guidelines. Detailed protocols and procedures are available in the Supplementary Material.

### 2.7 *In vitro* degradation

All nanofibrous scaffolds were sectioned into geometrically identical specimens. The samples were subjected to *in vitro* degradation studies by immersion in 6 mL of phosphate-buffered saline (PBS) containing 120 μL of dual antibiotic solution. This degradation process was conducted in a temperature-controlled orbital shaker maintained at 37 °C with constant agitation at 100 rpm for predetermined time intervals to simulate physiological conditions. Following degradation, specimens underwent sequential post-processing: rinsing with deionized water, lyophilization in a freeze-dryer, and subsequent gravimetric analysis. Weight loss percentage was calculated using the following gravimetric equation:

$$\text{Weight loss }\left(\%\right)=\frac{w_{0}-w_{d}}{w_{0}}\times 100\%$$ (1)

Where W_0_ represents the initial dry mass and W_d_ denotes the post-degradation dry mass. Triplicate measurements were performed for statistical reliability. The pH evolution of the PBS medium post-degradation was monitored using a calibrated digital pH meter (PHS-3C, INESA Scientific Instrument Co., China). All nanofibrous scaffolds before and after degradation for different time periods were characterized using SEM, FTIR, TGA, dynamic hybrid rheometer(DHR) and gel permeation chromatography (GPC). Detailed experimental procedures and supplementary data are provided in the Supplementary Material.

### 2.8 Statistical analysis

Statistical analysis was performed using Origin 9.5. One-way analysis of variance (ANOVA) was used for intergroup comparisons. A P-value of less than 0.05 was considered statistically significant.

## 3 Results and discussion

### 3.1 Morphological and structural characterization

**FIGURE 1:**
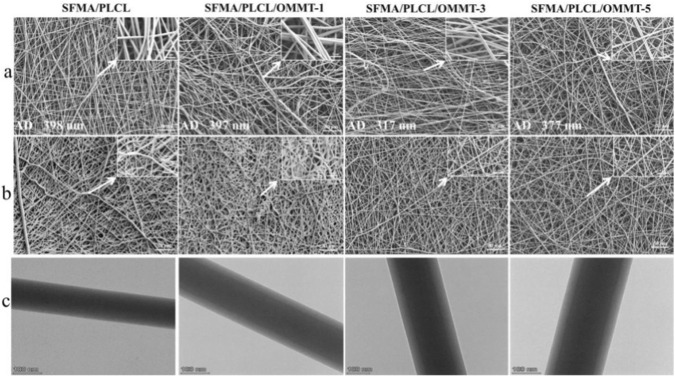
SEM images of SFMA/PLCL/OMMT nanofibrous scaffolds before and after treatment with 75 % ethanol vapor **(a,b)** and TEM images **(c)**.

Figure 1a displays the surface morphology of nanofibrous scaffolds before and after treatment with 75 vol% ethanol vapor. Pristine SFMA/PLCL nanofibers have smooth surfaces with an average diameter of 398 nm. As OMMT content increases, the fiber diameter first increases and then decreases, with slight irregular protrusions due to OMMT aggregation. Ethanol treatment induces swelling and partial inter-fiber fusion in pristine and 1 wt% OMMT scaffolds. However, scaffolds with 3 wt% and 5 wt% OMMT retain their fibrous structure, demonstrating the crucial role of OMMT in enhancing dimensional stability. Figure 1b shows that nanofibers without OMMT lack a core-shell structure, while nanofibers with OMMT form a distinct core-shell structure. At 1 wt% OMMT, the filler content is insufficient to form a stable internal framework, leading to nanofiber swelling. In contrast, ≥3 wt% OMMT results in a clearer core-shell structure, with platelets concentrated in the intermediate layer, significantly improving mechanical strength and dimensional stability (Hong et al., 2022).

**FIGURE 2:**
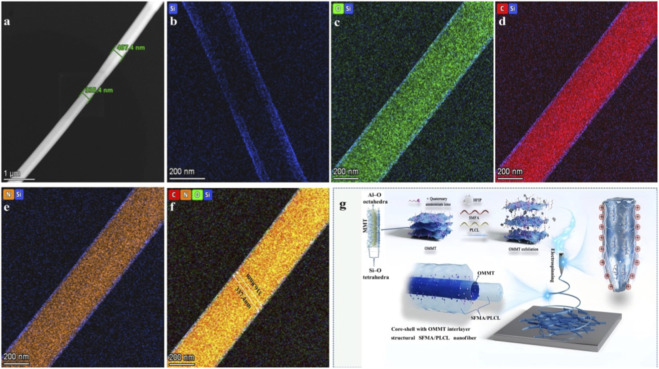
EDS and elements mapping images of SFMA/PLCL/OMMT-3 nanofiber: **(a)** photograph of nanofiber irradiated by a high-pressure electron beam (200 kV); **(b–f)** mapping images of different elements; **(g)** Formation mechanism of core-shell with OMMT interlayer nanofiber.

EDS mapping analysis (Figure 2) further confirms this architecture. Under 200 kV electron beam irradiation, the average nanofibrous diameter decreases from 407.4 nm to 335.4 nm due to partial melting of the outer layer (Figure 2a). Elemental mapping analysis (Figures 2b–f) reveal carbon (C) and nitrogen (N) concentrated within silicon (Si)-rich ring, while oxygen (O) is distributed throughout both inner and outer domains. Quantitative thickness measurements assign approximately 10 nm (outer shell) and 317 nm (inner core). Taking into account the interlayer spacing of OMMT (3.134 nm, Supplementary Figure S1) and the observed elemental gradients, the irradiated zone is described as a core-shell structure in which OMMT platelets form a discrete interfacial layer encasing an SFMA/PLCL core. Accordingly, the overall nanofiber adopts a hierarchical core-shell configuration with OMMT interlayer, while SFMA/PLCL occupies both the core and the shell. This architecture parallels our previous observations in SF/PLLA systems (Gu et al., 2024). The key to forming the above structure is that OMMT delamination in HFIP produces a stable, emulsion-like electrospinning solution. (Figure 2g). The present system produces thicker nanofibers at low OMMT levels. This is due to the greater chain flexibility of PLCL, which increases solution viscosity. As a result, nanofibers show greater ethanol-induced swelling during electrospinning (Duan et al., 2021).

**FIGURE 3:**
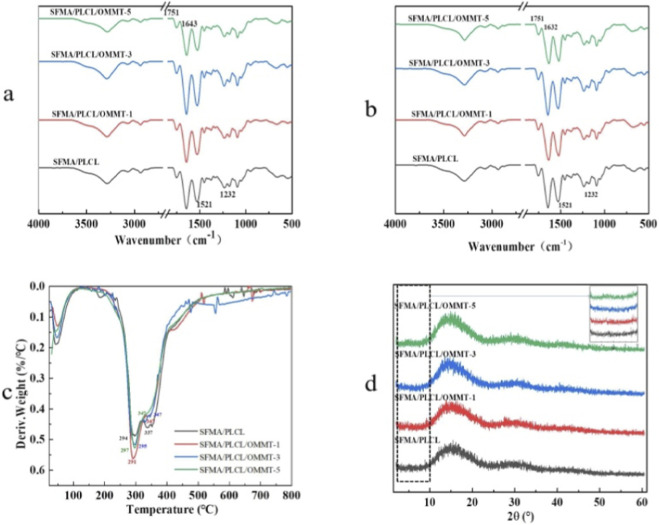
FTIR-ATR of SFMA/PLCL/OMMT nanofibrous scaffolds before and after treatment with 75% ethanol vapor **(a,b)**, DTG **(c)** and XRD **(d)** curves of nanofibrous scaffolds.

FTIR-ATR spectra (Figures 3a,b) show characteristic peaks at 1751 cm^-1^ (PLCL ester carbonyl) and at 1,643, 1,526, and 1,234 cm^-1^ (amide I-III of SFMA). These remain unchanged with OMMT incorporation, indicating no significant β-sheet formation or strong chemical interactions in as-spun fibers (Safdari et al., 2016). After ethanol treatment, red-shifts of amide I and II bands to 1,632 and 1,519 cm^-1^ indicate β-sheet formation. However, β-sheet content, calculated from amide I, decreases from 39% to 28% as OMMT content reaches 5 wt% (Supplementary Figure S2), likely that the quaternary ammonium ions released into the SFMA/PLCL matrix disrupt the formation of ordered hydrogen-bonding networks between SFMA chains by inducing electrostatic interference and steric hindrance, thereby suppressing the development of β-sheet crystalline structures (Milyaeva et al., 2025; Lu et al., 2014). DTG analysis (Figure 3c) shows pristine SFMA/PLCL exhibits two maximum degradation rate temperatures (T_d, max_) at 294 °C and 337 °C, corresponding to SFMA and PLCL, respectively (Zhang, 2010). In OMMT-containing scaffolds, the lower T_d, max_ remains essentially unchanged (291 °C–297 °C), but the higher T_d, max_ shifts to 347 °C (an increase of 10 °C), reflecting improved thermal stability of PLCL. In addition, the quaternary ammonium groups display greater thermal resilience when immobilized within the nanofibrous matrix than in their free state (Liu et al., 2022). XRD patterns (Figure 3d) reveal no new peaks or shifts with OMMT addition, indicating preserved SFMA structure. The disappearance of basal OMMT reflections (2θ = 2°–10°) confirms complete exfoliation and homogeneous dispersion of silicate layers in the composite. The uniformly dispersed OMMT nanosheets reinforce the fiber structure and hinder SFMA crystallization, confirming their dual role as structural and interfacial stabilizers.

### 3.2 Hydrophilicity, moisture permeability and mechanical properties

**FIGURE 4:**
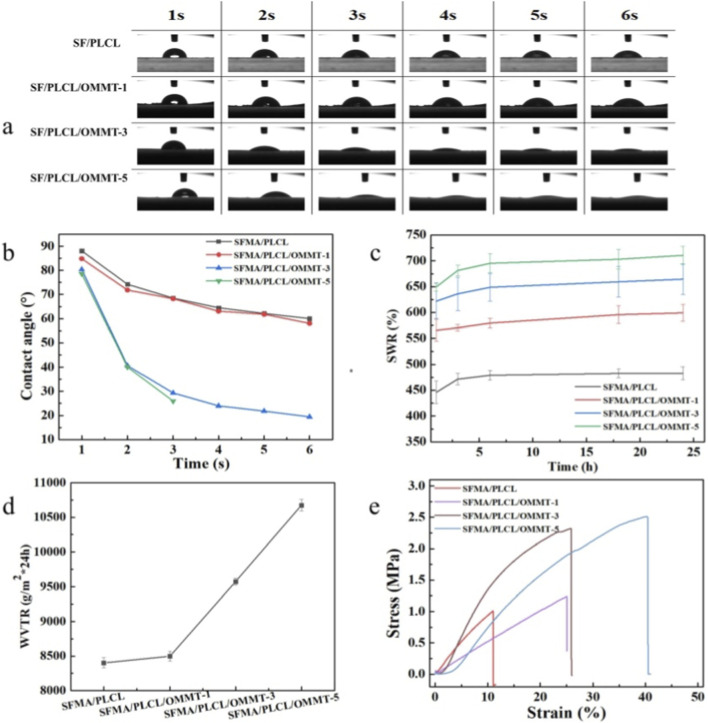
Hydrophility of SFMA/PLCL/OMMT nanofibrous scaffolds: photographs and curves of time-dependent water contact angles **(a,b)**; swelling water rate **(c)**; moisture vapor transmission rate **(d)**; mechanical properties **(e)**.

Figures 4a–e summarizes the hydrophilicity of the core-shell with OMMT interlayer nanofibrous scaffolds as assessed by static water contact angle measurements and swelling analyses. The initial contact angles of all scaffolds were <90°, with a rapid decay within the first 6 s. This decay became markedly steeper once the OMMT loading reached ≥3 wt%, accompanied by a monotonic increase in equilibrium swelling ratio. The improved hydrophilicity arises from two synergistic mechanisms. (1) Exfoliated OMMT layers release quaternary ammonium groups, acting as surfactants that reduce interfacial tension and enhance water uptake. Additionally, slight OMMT on the fiber surface contribute to wettability (Madejová et al., 2020). The OMMT nanosheets within the interlayer, rich in Si-O and Al-OH groups, readily form hydrogen bonds with water, facilitating permeation (Sabeti Dehkordi et al., 2015). As a result, moisture permeability (Figure 4d) increases significantly with higher OMMT content. This tunable hydrophilicity and permeability render the scaffolds promising for applications such as tissue engineering, smart wound dressings, and drug/growth factor delivery (Gao et al., 2021; Chen et al., 2021; Xu et al., 2024). Mechanical performance in the wet state (Figure 4e; Supplementary Table S1; Supplementary Figure 3S) further supports the applicability of these scaffolds in physiological environments. At 1 wt% OMMT, tensile strength remains similar to the pristine SFMA/PLCL, while elongation at break increases from 19.18% to 25.19%. At 3 wt%, tensile strength rises to 2.32 MPa, and at 5 wt%, both tensile strength and elongation at break reach 2.50 MPa and 40.45%, respectively. These enhancements stem from the reinforcing effect of OMMT within the nanofibrous interlayer, combined with the elasticity of PLCL, resulting in concurrent improvements in strength and flexibility (Yu et al., 2025; Duan et al., 2022). An optimal OMMT content enables fine-tuning of both hydrophilic and mechanical properties, expanding the potential of these nanofibrous scaffolds for advanced biomedical applications.

### 3.3 Antibacterial and anti-biofilm activity

**FIGURE 5:**
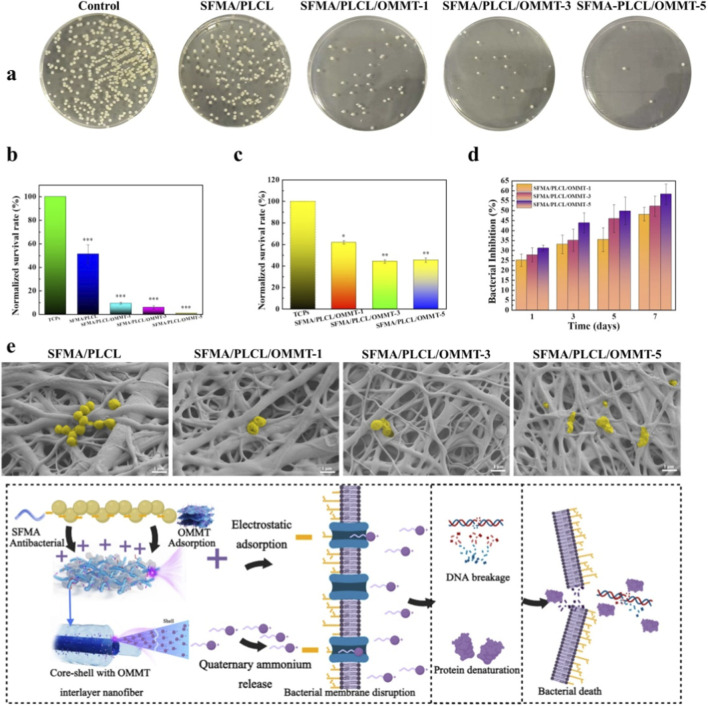
Antibacterial activity of SFMA/PLCL/OMMT nanofibrous scaffolds; macroscopic images of *Staphylococcus aureus* colonies on agar plates after treatment with nanofibrous scaffolds **(a)**; survival rate of *Staphylococcus aureus* co-cultured with nanofibrous scaffolds **(b)**; anti-biofilm effect co-cultured with nanofibrous scaffolds **(c)**; bacterial inhibition rate of *Staphylococcus aureus* in the sustained-release solution derived from the core-shell with OMMT interlayer nanofibrous scaffolds immersed in PBS over 1, 3, 5, and 7 days **(d)**; SEM images of *Staphylococcus aureus* cultured on nanofibrous scaffolds for 16 h **(e)**; Antibacterial mechanism **(f)**. Data are presented as mean ± SD (n = 3), ***p < 0.001, **p < 0.01, *p < 0.05.

Staphylococci are key pathogens in catheter-associated infections due to their ability to form biofilms (structured communities of sessile cells) on medical implants and damaged tissues (Jee et al., 2025; Gomes et al., 2025). The biofilms contribute to chronic infection persistence and heightened antimicrobial resistance (Singh et al., 2025). Thus, evaluating the antibacterial and anti-biofilm efficacy of nanofibrous scaffolds against *S. aureus* is of significant clinical relevance. SFMA/PLCL nanofibrous scaffolds exhibited moderate antibacterial (42%) and anti-biofilm (59%) activities. Incorporation of OMMT markedly enhanced both effects in a concentration-dependent manner, with antibacterial efficiency increasing from 95% to 99% and anti-biofilm activity from 75% to 77% as OMMT content rose from 1 wt% to 5 wt% (Figures 5b,c). The antibacterial activity of sustained-release solutions derived from the core-shell with OMMT interlayer nanofibrous scaffolds immersed in PBS over 1, 3, 5, and 7 days was evaluated (Figure 5d). All sustained-release solutions containing OMMT nanofibrous scaffolds exhibited enhanced antibacterial performance with increasing release time. Furthermore, the antibacterial activity of the sustained-release solutions improved with higher OMMT content. This controlled, time-dependent release originates from the core-shell structure with OMMT interlayer, where quaternary ammonium salts first dissociate from the OMMT and subsequently diffuse through the fiber shell before being gradually released into the medium. Microscopic morphological observations (Figure 5e) further revealed that bacterial cells adhered to SFMA/PLCL scaffolds retained their characteristic intact grape-like clusters. In contrast, cells on scaffolds with 1 wt% and 3 wt% OMMT displayed significant deformation and shrinkage, while those in contact with 5 wt% OMMT-containing scaffolds underwent complete structural disintegration, appearing as amorphous masses.

These findings confirm that incorporating OMMT significantly enhances both antibacterial and anti-biofilm activities in a concentration-dependent manner, providing durable antimicrobial efficacy. This improvement is driven by a synergistic interaction between the nanofibrous matrix and OMMT (Figure 5f). The high surface area of the nanofibers, the intrinsic antimicrobial properties of SF (Schäfer et al., 2022), and the sustained release of quaternary ammonium group work together to promote bacterial adhesion through electrostatic attraction and hydrophobic interactions. OMMT’s positively charged surface (Supplementary Figure 1S) creates localized nutrient deprivation zones, accelerating bacterial aggregation and making them more vulnerable (Mokhtar et al., 2023). The released cationic quaternary ammonium groups disrupt bacterial membranes and interfere with metabolism, leading to bacterial death (Onofre-Renteria et al., 2025). Moreover, the continuous release of these cationic groups from the OMMT nanofiber composite ensures long-term antibacterial activity by maintaining electrostatic targeting and hydrophobic membrane penetration, which reduces the risk of resistance development (Chen et al., 2024). This dual-action mechanism achieves over 99% inhibition of *S. aureus* and offers a strategy against multidrug-resistant bacteria. It effectively suppresses biofilms (>75% efficiency) while maintaining sustained antimicrobial activity.

### 3.4 Cytocompatibility

**FIGURE 6:**
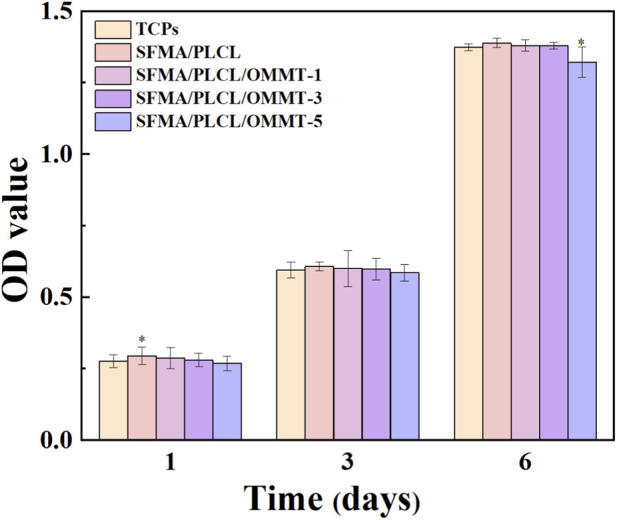
Absorbance values reflecting the cytocompatibility of SFMA/PLCL/OMMT nanofibrous scaffolds (OD 492 nm). Data are presented as mean ± SD (n = 3), ***p < 0.001, **p < 0.01, *p < 0.05.

The cytocompatibility of the nanofibrous scaffolds was assessed using human umbilical vein endothelial cells (HUVECs) over a 6-day culture period (Figure 6). A slight concentration-dependent decline in cell viability was observed with increasing OMMT content. Scaffolds containing 5 wt% OMMT showed a statistically significant reduction in viability compared to the tissue culture plate (TCP) control after 6 days; however, Compared with TCP, the absorbance remains as high as 94.4%. Despite this marginal decrease, all scaffold groups maintained viability above the 90% threshold commonly considered indicative of good cytocompatibility. This suggests that the incorporation of OMMT, even at higher concentrations, does not exert significant cytotoxic effects on endothelial cells. The observed biocompatibility stems from the favorable surface properties of the SFMA/PLCL matrix, which promote cell adhesion and proliferation. Controlled release of OMMT-derived components further minimizes acute cellular stress.

### 3.5 Degradation behavior and mechanism

**FIGURE 7:**
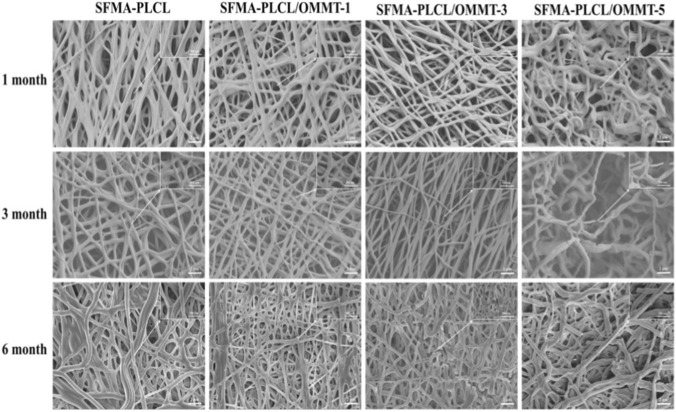
SEM images of SFMA/PLCL/OMMT nanofibrous scaffolds after 1, 3 and 6 months of degradation.

**FIGURE 8:**
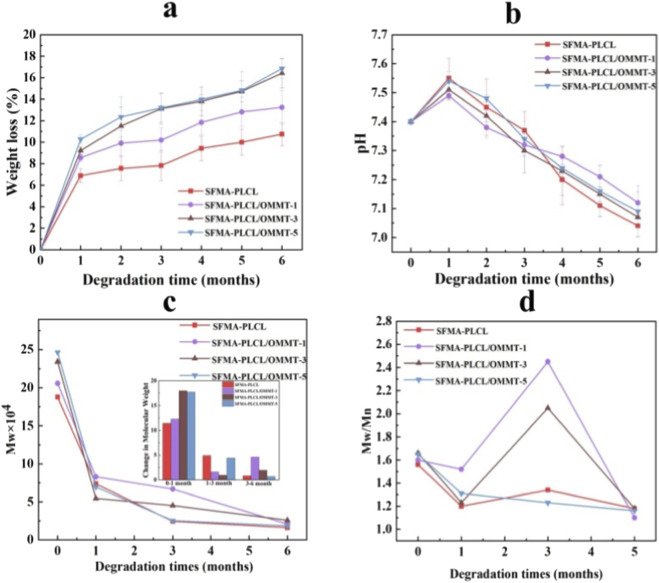
Weight loss **(a)** of SFMA/PLCL/OMMT nanofibrous scaffolds after 1, 3 and 6 months of degradation; pH changes **(b)** of PBS buffer solution with nanofibrous scaffolds after degradation for different time periods; Molecular weight (Mw) changes **(c)** and PDI **(d)** of PLCL after degradation for different time periods in PBS.

**FIGURE 9:**
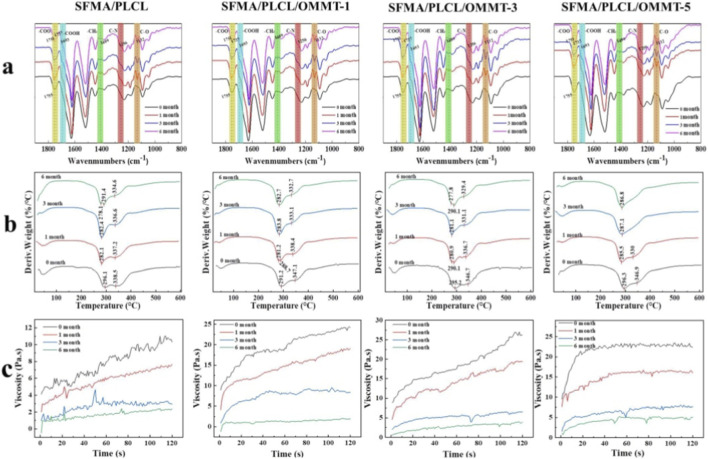
FTIR-ATR spectra **(a)**, DTG curves **(b)** and viscosity change curves **(c)** of SFMA/PLCL/OMMT nanofibrous scaffolds before after degradation for different time periods in PBS.

The degradability of biomaterials is critical in tailoring scaffold performance for regenerative medicine. The degradation behavior of a core-shell with OMMT interlayer SFMA/PLCL nanofibrous scaffolds was systematically evaluated in PBS over a 6-month period (Figures 7–9). Figure 7 reveals SEM images of nanofibrous scaffolds after degradation for different time periods in PBS. After 1 and 3 months of degradation, the pristine SFMA/PLCL nanofibers and those containing 1 wt% and 3 wt% OMMT exhibited only slight swelling, whereas the nanofibers with 5 wt% OMMT demonstrated significant curling, swelling and partial structural rupture. After 6 months of degradation, the pristine SFMA/PLCL nanofibers displayed localized thinning, the 1 wt% OMMT samples exhibited sporadic fracture points, the 3 wt% OMMT nanofibers experienced increased fragmentation, and the 5 wt% OMMT nanofibers underwent substantial contraction and extensive rupture across most regions. The results show the presence of OMMT accelerates the degradation process, particularly at higher concentrations. Interestingly, despite visible fragmentation, all nanofibers retained their fibrous morphology, indicating that degradation initiated internally while preserving superficial structure, especially in low-OMMT scaffolds. This phenomenon is largely governed by the 8:2 ratio of SFMA to PLCL in the nanofibrous matrix. Typically, the degradation rate of SF in PBS is significantly slower than that of PLCL. This slower rate can be attributed to the β-sheet conformation of SF induced by ethanol treatment, which results in higher crystallinity, as well as the greater hydrolytic stability of amide bonds in PBS compared with ester bonds (Zhang et al., 2011; Mndlovu et al., 2024).

Figure 8a presents the weight changes of nanofibrous scaffolds after incubation in PBS for up to 6 months. A clear trend of increasing weight loss is observed with higher OMMT content, indicating that OMMT promotes scaffold degradation. Nevertheless, the overall weight loss remains relatively limited, primarily due to the slow degradation rate of SFMA (Zhang et al., 2011). The weight loss of scaffolds containing 3 wt% and 5 wt% OMMT is nearly identical, which contrasts with the more pronounced morphological degradation observed in SEM images. This inconsistency may stem from the experimental procedure, wherein the degradation products were filtered using filter paper, dried, and weighed. It is possible that some soluble oligomeric compounds were adsorbed onto OMMT owing to its high adsorption capacity, thereby leading to an underestimation of the actual weight loss. Furthermore, the strong interfacial interactions between OMMT and the polymer matrix may also contribute to the retention of degraded fragments within the scaffold structure, thereby affecting the measured mass loss. During the degradation process, weight loss primarily results from the diffusion and dissolution of soluble oligomeric compounds into the degradation medium, which originate from the hydrolytic cleavage of polymer chains (Wang et al., 2008). For all nanofibrous scaffolds, the pH variations (Figure 8b) of the degradation solution initially increase and subsequently decrease over time, with no significant differences. This phenomenon can be primarily attributed to the degradation products of SFMA, which mainly consist of polypeptides or amino acids containing amino (-NH_2_) and hydroxyl (-OH) groups capable of neutralizing the carboxyl groups (-COOH) produced from PLCL degradation. This inherent buffering mitigates localized acidosis, reducing the risk of inflammation or cytotoxicity in physiological environment (Elmowafy et al., 2019).

The molecular weight of the PLCL component in nanofibrous scaffolds before and after hydrolytic degradation was analyzed by GPC using THF as the mobile phase, in which SFMA is insoluble (Figures 8c,d). Unexpectedly, in the undegraded nanofibrous scaffolds, the apparent molecular weight of PLCL increased with rising content of OMMT. This trend is counterintuitive but can be explained by strong intermolecular interactions such as hydrogen bonding between SFMA and PLCL, which limit the solubilization of PLCL in THF, resulting in underestimated GPC values. Previous studies have confirmed that such interactions can reduce polymer extractability (Zhang et al., 2011). The incorporation of OMMT disrupts these interactions, as supported by FTIR data showing a reduction in hydrogen bonding intensity with increasing OMMT content. This promotes the release of more PLCL chains into the THF solution, thereby increasing the apparent molecular weight measured. To confirm this hypothesis, undegraded nanofibrous scaffolds were immersed in THF for 7 days, and the mass of the dissolved fraction was quantified (Supplementary Figure 4S). The results clearly showed a positive correlation between OMMT content and solubilized PLCL, validating that the incorporation of OMMT weakens the SFMA-PLCL interactions and enhances polymer extractability. During degradation, the Mw of PLCL in all nanofibrous scaffolds exhibited a sharp initial decline, followed by a slower rate of decrease. After 6 months, nanofibrous scaffolds containing 3 wt% and 5 wt% OMMT showed the most pronounced molecular weight loss, indicating that OMMT accelerates the hydrolytic cleavage of PLCL chains. This acceleration may arise from the disruption of hydrogen bonds and the facilitation of water molecule penetration into the fiber matrix. Polydispersity index (PDI) analysis further elucidated the degradation behavior. After 3 months, nanofibers without OMMT and with 3 wt% OMMT displayed broader molecular weight distributions compared to those with 1 wt% and 5 wt% OMMT. The results suggest heterogeneous degradation with accumulation of lower molecular weight fragments. Only a small fraction of these products was released into PBS, indicating that weight loss lags behind molecular weight reduction, a typical feature of early-stage bulk erosion.

FTIR-ATR spectra before and after degradation (Figure 9a) reveal consistent spectral changes across all nanofibrous scaffolds. After degradation, the characteristic ester carbonyl (-COO) absorption peak at 1751 cm^-1^ split into two peaks at 1755 cm^-1^ and 1737 cm^-1^ corresponding to -COO of PLA chain segments and PCL chain segments, respectively. This splitting reflects chain scission and the resulting differentiation in chemical environments between PLA and PCL units (Przybysz-Romatowska et al., 2020). New absorption peaks appeared at 1,693 cm^-1^, 1,409 cm^-1^, 1,132 cm^-1^, and 1,256 cm^-1^ after degradation, corresponding to the absorption of -COOH groups generated from PLCL hydrolysis, enhanced -CH_2_ bending vibrations, C-O stretching of ester bonds, and C-N stretching vibrations from degraded SFMA, respectively (Erdal et al., 2020). Amide I (1,627 cm^-1^), II (1,523 cm^-1^), and III (1,235 cm^-1^) bands, characteristic of β-sheet structures in SFMA, remained positionally stable. However, secondary structure analysis of the amide I region indicated a significant increase in β-sheet content after degradation, particularly in scaffolds containing 5 wt% OMMT (Supplementary Figure 5S). This increase is attributed to hydrolytic cleavage of disordered (random coil and α-helix) domains, promoting molecular rearrangement and formation of hydrogen bond driven β-sheet conformations. Broadening of the 1,235 cm^-1^ band further supports enhanced intermolecular hydrogen bonding (Takahashi et al., 2007).

Figure 9b presents the DTG curves of the nanofibrous scaffolds after degradation. Following degradation, all nanofibrous scaffolds exhibited a reduction of approximately 10 °C in the T_d, max_ of the first decomposition stage (200 °C–315 °C) owing to the hydrolytic degradation of SFMA leading to the consequent reduction in molecular weight. The T_d, max_ of the second decomposition stage (315 °C–400 °C) corresponding to the thermal degradation of PLCL, remained nearly unchanged in the pristine SFMA/PLCL nanofibrous scaffolds. However, upon incorporation of OMMT, a significant decrease of approximately 10 °C in the T_d, max_ of this stage was observed after degradation. Furthermore, the peak area progressively decreased with increasing degradation time, especially in 5 wt% OMMT scaffolds, indicating progressive loss of PLCL due to soluble oligomer or monomers formation and diffusion into the medium. These results further confirm that the incorporation of OMMT accelerates the degradation of the polymer matrix, particularly the PLCL component.

Rheological measurements (Figure 8c) provided further insight. Solutions of nanofibrous scaffolds dissolved in a 9.3 M lithium bromide(LiBr)/THF mixture (v/v = 1:1) exhibited markedly higher viscosity in OMMT-containing nanofibibrous scaffolds prior to and after short-term degradation. This enhancement arises from the high aspect ratio and surface functionality (Si-O, Al-OH) of OMMT nanosheets, which promote hydrogen bonding and physical entanglement with polymer chains in solutions, forming a pseudo-crosslinked network that limits chain mobility (Guo et al., 2018). With increasing degradation time, viscosity decreased across all nanofibrous scaffolds, reflecting molecular weight reduction and diminished chain entanglement (Van Dijk et al., 1983). The decline in viscosity was more evident in high-OMMT scaffolds, further suggesting that OMMT accelerates degradation kinetics.

**SCHEME 2:**
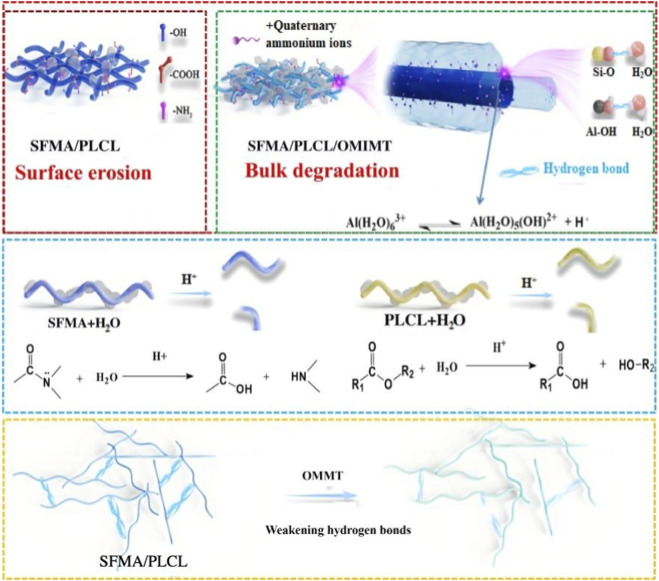
Mechanistic influence of OMMT incorporation on the degradation behavior of SFMA/PLCL nanofibers

The aforementioned series of characterization results clearly indicate that the incorporation of OMMT enhances the degradation behavior of SFMA/PLCL nanofibrous scaffolds in PBS. Moreover, the degradation rate of SFMA within the nanofibers is slower than that of PLCL, which aligns with previously reported findings (Zhang et al., 2011). In general, the degradation of electrospun nanofibrous scaffolds is governed by two predominant mechanisms: bulk degradation and surface erosion. Bulk degradation occurs when the scaffold exhibits high diffusivity, allowing water molecules to penetrate deeply into the polymer matrix and hydrolyze chains in the interior of the nanofibers. This internal hydrolysis often leads to structural collapse and facilitates the diffusion of monomers or oligomers. In contrast, surface erosion takes place when water permeates only a superficial layer of the nanofibers before initiating hydrolytic degradation. In such cases, the release of degradation products is primarily confined to the surface or erosion front, with negligible diffusion from the interior. Typically, the rate of degradation via the bulk degradation mechanism is significantly greater than that observed in surface erosion (Xu et al., 2024). With the incorporation of OMMT, the degradation mechanism of SFMA/PLCL nanofibrous scaffolds appears to transition from surface erosion to bulk degradation. This transformation can be attributed to the following factors (Scheme 2): (1) Upon immersion in PBS, the SFMA component containing hydrophilic functional groups such as -NH_2_, -OH, -COOH tends to adsorb onto the nanofibrous surface via hydrogen bonding interactions. Previous studies have demonstrated that in SF/PLCL blend nanofibers, SF is predominantly localized at the fiber surface (Zhang, 2010). Upon contact with water, the β-sheet crystalline structure of SFMA impedes the inward diffusion of water molecules, thereby limiting the hydrolysis of the ester bonds in PLCL. As a result, chain scission occurs primarily at the fiber surface, and the degradation process is dominated by surface erosion. This phenomenon is supported by SEM analysis, which shows that even after 6 months of immersion, the fibers display only slight swelling and localized thinning. With the introduction of OMMT, the resulting nanofibers adopt core-shell architecture with OMMT interlayer. Upon exposure to PBS, the Si-O and Al-OH groups present in the OMMT nanosheets of interlayer can establish strong hydrogen bonds with water molecules, thereby enhancing water ingress into the nanofiber core and promoting nanofiber swelling. Moreover, quaternary ammonium ions released from exfoliated OMMT, which possess high hydrophilicity and surface activity, can partially diffuse into the polymer matrix, further facilitating water penetration. As evidenced in Figures 4a–c, the inclusion of OMMT significantly increases both water uptake and swelling capacity of the nanofibers. (2) The enhanced water infiltration promotes more uniform interaction between water molecules and the polymer matrix, resulting in more extensive hydrolytic cleavage of ester and amide bonds. Additionally, Surface- or interlayer-associated water and cations (e.g., Al^3+^) in OMMT can promote water dissociation, generating H^+^ ions that catalyze the hydrolytic cleavage of ester and amide bonds (Bhattacharyya and Gupta, 2008). This leads to a reduction in molecular weight and ultimately results in bulk degradation, characterized by internal fiber fragmentation. (3) The incorporation of OMMT weakens intermolecular interactions between polymer chains, such as hydrogen bonding and van der Waals forces, allowing water molecules to more easily penetrate the matrix. This enhanced water accessibility accelerates hydrolytic cleavage of ester and amide bonds, particularly in hydrophilic or semi-crystalline regions, thereby promoting bulk degradation. Therefore, the incorporation of OMMT not only alters the structural characteristics of the nanofibers, favoring a core-shell configuration, but also accelerates the degradation process by facilitating a shift toward bulk degradation. By adjusting the OMMT content, the degradation rate of the nanofibers can be finely controlled, offering a powerful tool for tailoring scaffold resorption profiles to match specific biomedical needs. This OMMT-mediated degradation mechanism offers a significant advantage over conventional fixed or passive systems. It provides a foundation for designing smart, bioresponsive materials. Such materials have potential applications in tissue engineering, wound healing, and controlled drug delivery.

## 4 Conclusion

The current study successfully fabricated and characterized innovative core-shell with OMMT interlayer SFMA/PLCL nanofibrous scaffolds. The unique core-shell design and OMMT incorporation significantly enhanced scaffold mechanical integrity, thermal stability, hydrophilicity, moisture permeability, and antibacterial properties. In-depth degradation analyses demonstrated that higher OMMT concentrations accelerate scaffold degradation, shifting the mechanism from surface erosion to bulk degradation. This tunable degradation capability, combined with robust antibacterial efficacy, positions these nanofibrous scaffolds as highly promising candidates for advanced biomedical applications, particularly in controlled drug delivery systems, wound care, and tissue regeneration scaffolds. Future research could focus on optimizing scaffold composition further and evaluating *in vivo* performance to facilitate clinical translation. Overall, this study provides valuable guidance for the strategic design and development of multifunctional nanofibrous biomaterials through controlled OMMT incorporation.

## Data Availability

The original contributions presented in the study are included in the article/Supplementary Material, further inquiries can be directed to the corresponding authors.
