# Supplementary material for: Biomimetic core–shell nanofibrous scaffolds with an organic montmorillonite interlayer for tissue engineering
Source: Front Bioeng Biotechnol. 2026 May 14;14:1836027. doi: 10.3389/fbioe.2026.1836027 (PMC13215998; doi:10.3389/fbioe.2026.1836027)
Supplement: Supplementary file 1 [file Supplementaryfile1.docx]

**Supporting information for**

**Biomimetic Core–Shell Nanofibrous Scaffolds with an Organic Montmorillonite Interlayer for Tissue Engineering**

Xiangrui Mao^a^, Ruobing Cheng^b^, Jian Zhang^a^, Yuxiang Yao^a^, Nan Jiang^a^, Jinbiao Zhang^d^, Tanlong Liu^a^, Zhirang Hu^a^, Yutong Liu^a^ ,, Zhiyong Yan^a^, Chengwei Yang^c^* Anlin Yin^a*^, Kuihua Zhang^a*^

^a^College of Materials and Textile Engineering, Jiaxing University, Zhejiang 314001, China

^b^Analytical and Testing Center, Jiaxing University, Jiaxing, Zhejiang 314001, China

^c^Department of Orthopaedics, The 940th Hospital of Joint Logistics Support Force of PLA, Lanzhou, Gansu, 730050, China

^d^ College of Materials Science and Engineering, Hunan University of Technology

**2.2 Fabrication of nanofibrous scaffolds**

**2.2.1 Fabrication of OMMT**

A certain amount of 10 wt% tetradecyl dimethyl benzyl ammonium chloride were added into Na^+^-MMT suspension of 5 wt% and reacted at 60 ^o^C for 6 h with continue stirring. The reactant was filtered and then washed with hot 50 % ethanol aqueous solution. The obtained product was frozen drying, grinded, and passed through a 200 mesh sieves and characterized by XRD and Zeta Potential

**2.2.2 Preparation of SFMA**

Raw silk was degummed three times with 0.5 wt % Na_2_CO_3_ solution at 100 °C for 30 min each and then washed with distilled water as well as dried to obtain degummed SF. 5g degummed SF was dissolved in 25 mL 9.3M LiBr solutions, and GMA of 571mM was slowly dropped into the mixture and then stirred at 60℃ for 3h. The prepared solution was dialyzed with a cellulose tubular membrane (250−7u; Sigma, St, Louis, MO, USA) in distilled water for 3 days at room temperature, and then ﬁltered as well as lyophilized to obtain SFMA sponges

**2.4 Evaluation of nanofibrous scaffolds properties**

**2.4.1 Mechanical properties**

Mechanical properties of all nanofibrous scaffolds were tested in a wet state. They were soaked in phosphate-buﬀered saline (PBS) for 30 min before measurement. The tensile properties of nanofibrous scaffolds were tested by a mechanics properties tester (H5K−S, Hounsﬁeld, England) at an elongation speed of 10 mm/min and 5 parallels for each sample.

**2.4.2 Swelling properties**

All samples were soaked in deionized water at 37 ^o^C and then taken out to absorb surface water with filter paper at the specified time. The weight of nanofibrous scaffolds before and after swelling was recorded as M_t_ and M_0_, respectively. The swelling water rate (SWR) was computed using the following equation and averaged from triplicate specimens.

$\mathrm{SWR}\left( \% \right)=\frac{M_{t-}M_{0}}{M_{0}}\times100\% (1)$

**2.4.3 Water vapor permeability**

The water vapor transmission rate (WVTR) was determined using the gravimetric method in accordance with ASTM E96 standard. Specifically, the test specimen was hermetically sealed on a permeation cup containing deionized water, which was then placed in a controlled environment maintained at 38±0.5°C and 50±2% relative humidity. The tests were carried out using YG601 moisture permeability equipment (Ningbo Textile Instrument Factory, China). The WVTR value, expressed in grams per square meter per 24 hours (g/(m²·24h)), was calculated based on the measured mass change over a 1-hour period using the following formula

$$WVTR=\frac{24\times(m_{1}-m_{2})}{s} (2)$$

where m_1_ and m_2_ denote the weight change of deionized water in the test assembly in 1 h, and S is the area of the samples

**2.5 In vitro antimicrobial and anti-biofilm assays**

**2.5.1 Antimicrobial properties**

Antibacterial performance was assessed using the shaking method, as outlined in GB/T 20944.3–2008. 10 mg sheared nanofibrous scaffolds were placed in a 50 mL centrifuge tube, sterilized with ultraviolet lamp for 2 h before introducing bacteria. The bacteria of Gram-negative *E. coli* and Gram-positive *S. aureus* were cultured in nutrient broth and shaken at 37 ^o^C overnight. The absorbance of the initial bacterial solution at 630 nm between 0.1 and 0.2 is equal to 0.5 Mcfarland standards of bacterial inoculums (10^8^ CFU/mL) and then diluted 10^5^ CFU/mL with PBS, respectively. A 10 mL bacterial inoculum was added into centrifuge tube and cultured at 37 ^o^C for 16 h. 100 μL cultured bacterial fluid was removed and then gradient diluted to 10^2^ CFU/mL with PBS. 100 μL diluent was coated on agar plate and then incubated at 37 ^o^C for 24 h, the number of colonies was counted and no nanofibrous scaffolds was inoculated with bacteria as control group. The percentage of bacterial inhibition (σ_bi_) was calculated by the colony count with and without nanofibrous scaffolds using the following equation:

$$Bacterial inhitbition\left( \sigma_{bi} \right)\%=\left( 1-\frac{w_{sample}}{w_{control}} \right)*100\% (3)$$

where w_control_ and w_sample_ were the colony count of with and without scaffolds after 16 h, respectively, Each value was averaged from triplicate specimens

The nanofibrous scaffolds after coculture for 16 h were washed three times with PBS and fixed with 2.5 % glutaraldehyde aqueous solution at 4 ◦C for 2 h. The fixed nanofibrous scaffolds were washed three times with PBS and dehydrated in graded ethanol solution (30, 50, 70, 80, 90, 95 and 100%) and then finally frozen drying for 24 h. The bacteria morphology was obtained by SEM.

**2.5.2 Anti-biofilm performance**

Sterilized specimens (three replicates per group) were aseptically placed into a 24-well culture plate, with sterile culture medium serving as a control. Following rehydration with phosphate-buffered saline (PBS), 200 μL of standardized bacterial suspension (1×10⁶ CFU/mL in PBS) was inoculated into each well. Biofilm formation was encouraged by static incubation at 37°C for 12 hours. After incubation, the planktonic bacteria were carefully removed, and each well was replenished with 200 μL of fresh nutrient broth, followed by a subsequent 12-hour metabolic activation period at 37 °C. Adherent biofilms were then washed three times with pre-warmed PBS (37 °C) to remove any non-adherent cells. The metabolic activity of the biofilms was quantified using the MTT colorimetric assay. Biofilm viability was assessed by normalizing the absorbance values of the experimental groups to those of the untreated controls, which were considered to represent 100 % metabolic activity.

**2.5.3 Antimicrobial properties of slow-release solution**

The sterilized samples were incubated in PBS and placed in a 37 °C shaking incubator of 80 rpm. On days 1, 3, 5, and 7, the collected supernatants were sealed and stored at 4°C. After the 7-day collection period, the bacterial suspension was diluted to 10⁷-10⁸ CFU/mL. In a sterile 96-well plate, 100 μL of the diluted bacterial suspension was added to each of 36 wells. Subsequently, 100 μL of supernatant from different time points (1, 3, 5, and 7 days) was added to the corresponding wells, with control groups (bacteria-only) included. The plate was incubated at 37°C for 24 hours. Bacterial growth was evaluated by measuring the optical density at 620 nm (OD620) using a microplate reader. The bacterial growth inhibition rate was calculated using the following formula

$Bacterial inhibition \left( wt\% \right)=(1-\frac{{EG}_{OD620}}{{CG}_{OD620}}$)×100wt% (4)

Where EGdenotes theExperimental Group and CG denotes the Control Group.

**2.6 Cytocompatibility evaluation**

Nanofibrous scaffolds were sterilized using 75 % ethanol vapor for 4 hours, then immersed in Dulbecco’s Modified Eagle Medium (DMEM) at a ratio of 1 g : 10 mL. The scaffolds were incubated at 37 °C with gentle agitation at 50 rpm for 48 hours. The resulting extract was subsequently filtered through a 0.22 μm membrane for use in cell culture experiments. HUVECs were seeded at a density of 1×10⁵ cells per well in a 24-well plate. Following a 4-hour adhesion period, the culture medium was replaced with DMEM supplemented with 10 % fetal bovine serum (FBS) for routine maintenance. Subsequently, 200 μL of the scaffold extract was added to each well. Three replicate wells were prepared for each condition, along with a blank control group. Cells were cultured for 1, 3, and 6 days. On days 1 and 3, the medium was refreshed by replacing it with 500 μL of DMEM containing 10 % FBS along with 200 μL of the scaffold extract. Cellular metabolic activity was assessed using the MTT assay. Relative cell viability was calculated based on absorbance measurements, and cytotoxicity was evaluated in accordance with ISO 10993-5 guidelines, whereby a reduction in cell viability of more than 30% is indicative of cytotoxic potential.

**2.7 In vitro degradation**

**2.7.1 Rotational Rheometer (DHR)**

Electrospun nanofibrous scaffolds before and after degradation were dissolved in a mixed solvent of 9.3 M LiBr and THF (volume ratio 1:1). The resulting solutions were subsequently used for rheological measurements. Rheological testing was performed using a rotational rheometer (MARS, Thermo Fisher Scientific) with parallel plate fixtures. A 2 mL solution of the degraded sample was placed on the testing platform. The measurements were conducted at a shear rate of 0.01 s⁻¹ and a temperature of 25 °C.

**2.7.2 Gel Permeation Chromatography (GPC)**

In this experiment, chromatographic-grade tetrahydrofuran (THF) was used as the solvent to dissolve the degraded samples for 7 days at a concentration of 5 mg/mL. The mixed solution was thoroughly stirred using a magnetic stirrer and then filtered through a microporous membrane with the appropriate pore size. After filtration, the solution was allowed to stand for half an hour before use. The instrument was turned on, and a suitable chromatographic column was selected, ensuring all components were properly connected. System equilibration was performed by allowing the mobile phase to flow through the system at a low flow rate until stability was achieved. Sample acquisition parameters were set, and the samples were placed in the test tray for automatic testing. Upon completion of the test, the data were saved and analyzed using software to determine the molecular weight distribution and weight-average molecular weight, thereby obtaining the experimental results.

**3. Results and discussion**


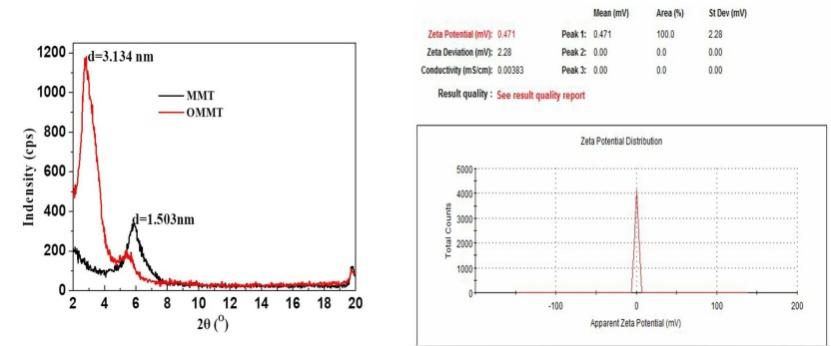


Fig.1S XRD curves and Zeta potential of MMT and OMMT


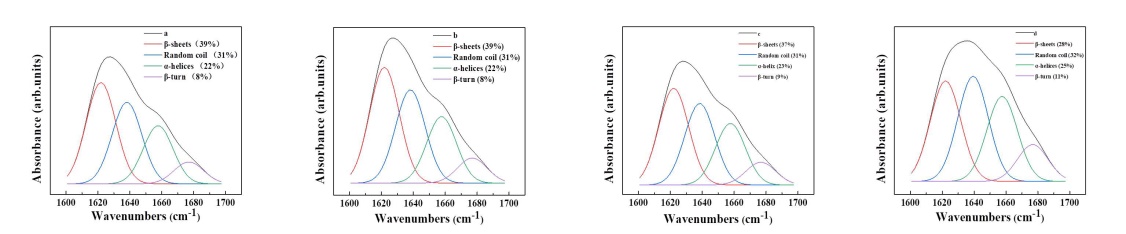


Fig. 2S Quantitative analysis of secondary structures of SFMA with Gaussian curve ﬁtting in FTIR-ATR spectra of nanofibrous scaffolds. (a) SFMA/PLCL; (b) SFMA/PLCL/OMMT-1; (c) SFMA/PLCL/OMMT-3; (d) SFMA/PLCL/OMMT-5


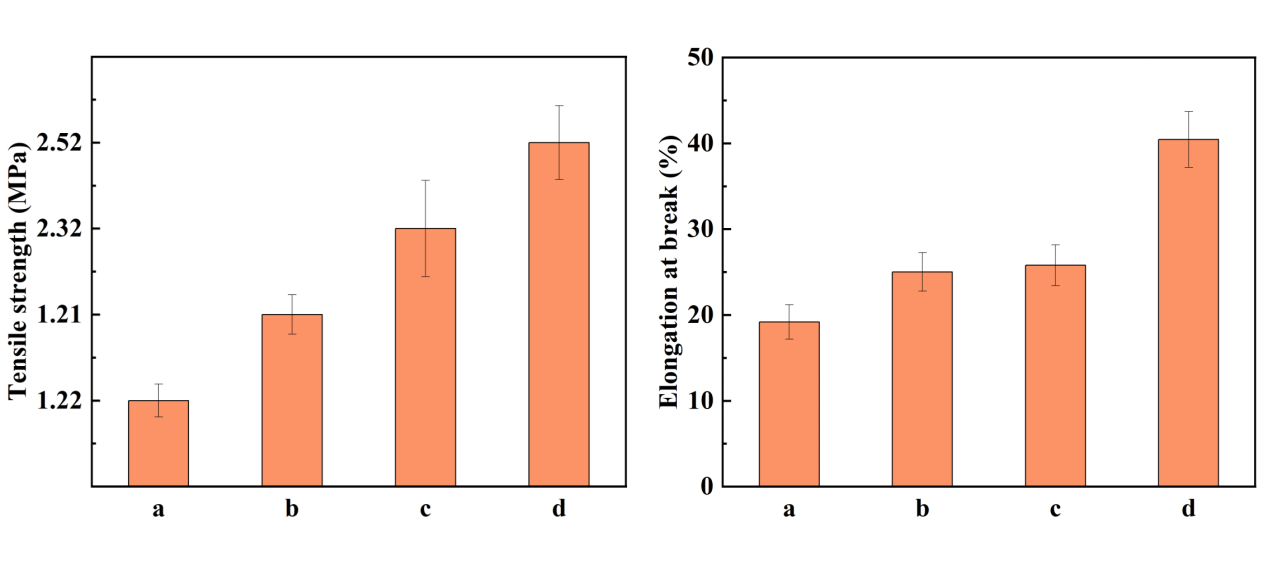


Fig. 3S Tensile strength and elongation at break of SFMA/PLCL/OMMT nanofibrous scaffolds. Data are presented as mean ± SD (n = 3). (a) SFMA/PLCL; (b) SFMA/PLCL/OMMT-1; (c) SFMA/PLCL/OMMT-3; (d) SFMA/PLCL/OMMT-5.

Tab. 1S Mechanical properties of nanofibrous scaffolds

|  | Specimen thickness  (mm) | Elongation at break  (%) | Tensile strength  (Mpa) |
| --- | --- | --- | --- |
| SFMA/PLCL | 0.063±0.003 | 19.18±2.41 | 1.22±0.11 |
| SFMA/PLCL/OMMT-1 | 0.049±0.008 | 25.09±0.11 | 1.24±0.47 |
| SFMA/PLCLOMMT-3 | 0.107±0.011 | 25.98±1.11 | 2.32±0.08 |
| SFMA/PLCL/OMMT-5 | 0.102±0.008 | 40.45±7.15 | 2.50±0.17 |


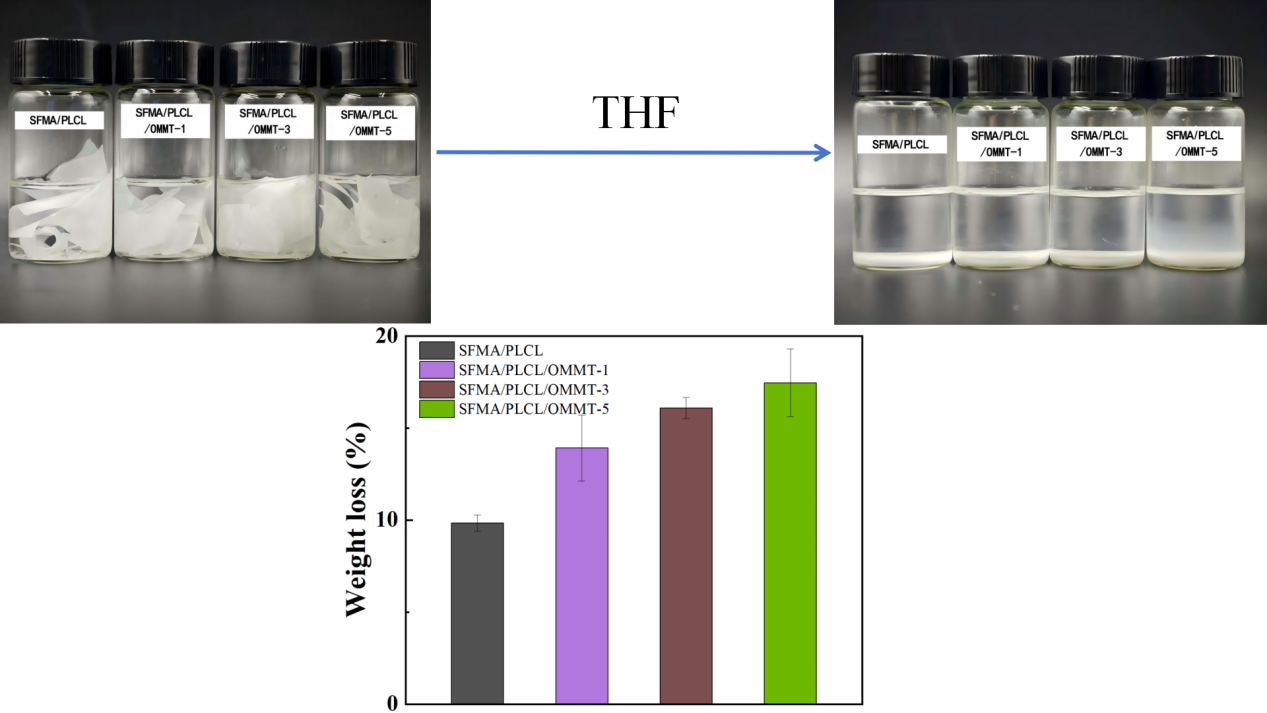


Fig. 4S Morphological changes (A) and weight loss (B) of nanofibrous scaffolds dissolved in THF Over a 7-Day Period (a) SFMA/PLCL; (b) SFMA/PLCL/OMMT-1; (c) SFMA/PLCL/OMMT-3; (d) SFMA/PLCL/OMMT-5


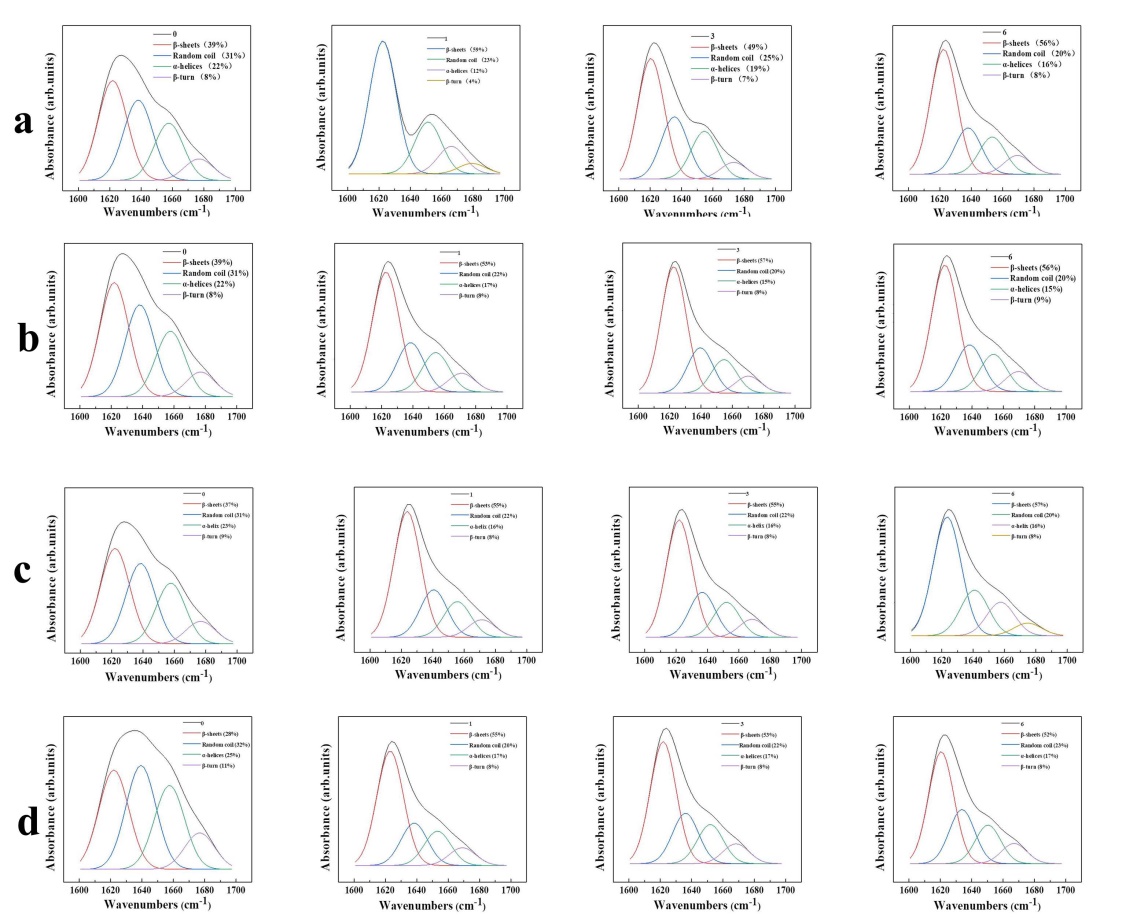


Fig. 5S Quantitative analysis of secondary structures of SFMA with Gaussian curve ﬁtting in FTIR-ATR spectra of nanofibrous scaffolds before and after degradation for different time. (a) SFMA/PLCL; (b) SFMA/PLCL/OMMT-1; (c) SFMA/PLCL/OMMT-3; (d) SFMA/PLCL/OMMT-5
